# An evaluation of factors affecting the usefulness and sustainability of inter-pharmacist case conferences

**DOI:** 10.1186/s40780-026-00599-7

**Published:** 2026-06-15

**Authors:** Shun Tsujimura, Kanayuki Kitahara, Shin Tanaka, Tatsuya Isezaki, Ryohkan Funakoshi

**Affiliations:** Department of Pharmacy, Kameda Medical Center 929 Higashi-Cho, Kamogawa, Chiba 296-0041 Japan

**Keywords:** Pharmacist case conference, Continuing professional development, Clinical decision-making, Psychological safety

## Abstract

**Background:**

Inter-pharmacist case conferences, in which practitioners routinely discuss drug therapy, are expected to improve the validity of clinical judgements and provide educational benefits. However, evidence regarding their utility and factors associated with sustainability is scarce. Therefore, this study aimed to clarify the utility of a year-long inter-pharmacist case conference program at Kameda Medical Center (Kamogawa, Japan) and explore factors associated with its continuity.

**Methods:**

A cross-sectional study was conducted with 40 pharmacists who participated in inter-pharmacist case conferences initiated in November 2023. A 16-item questionnaire that incorporated the Attention, Relevance, Confidence, Satisfaction model and psychological safety perspectives was administered. Exploratory factor analysis was performed as the primary endpoint. As a secondary analysis, the association between the extracted factors and motivation for continuity was examined using multiple regression analysis. Furthermore, the impact of experience as a facilitator or case presenter on factor scores was evaluated using two-way analysis of variance.

**Results:**

Three factors were extracted from the questionnaire data with a cumulative contribution rate of 75.5%. Factor 1 was interpreted as confidence and professional growth through conferences, Factor 2 as improvement in clinical judgement and quality of drug therapy, and Factor 3 as promotion of communication and improvement of the environment for expressing opinions. The mean factor score was highest for Factor 2. Motivation for continuity was significantly associated with Factors 1 and 2. Case presenter experience was associated with higher scores for Factor 1, suggesting that case presentation may be related to perceived professional growth.

**Conclusions:**

Inter-pharmacist case conferences may be a valuable practice-based initiative related to clinical judgement, self-efficacy, and team communication. The study results suggest that perceived professional growth and practical application to clinical judgement may be important elements associated with sustainability. Overall, this study provides fundamental insights into the institutionalization and dissemination of inter-pharmacist conferences.

**Supplementary Information:**

The online version contains supplementary material available at 10.1186/s40780-026-00599-7.

## Background

In their daily practice, healthcare professionals are obligated to make numerous clinical judgements, particularly judgements regarding drug therapies. This involves a series of processes, including evaluating indications, selecting agents, determining dosages and administration routes, and assessing therapeutic effects and drug reactions [1–3]. Although these judgements are based on clinical guidelines and evidence, they are not always unequivocal and require flexible adaptation to each patient’s pathophysiology, comorbidities, and lifestyle. This inherent uncertainty means that decisions made using limited information are often prone to error [2]. Consequently, strategies to reduce judgement errors and improve the validity of clinical decisions are urgently needed. The biases influencing decision-making among healthcare professionals have been explored [3], and multidisciplinary conferences can reduce diagnostic errors [2]. Furthermore, in Western countries multidisciplinary conferences contribute to improved patient outcomes, including reduced mortality [4], prolonged overall survival [5], and shortened hospital stay [6].

Based on these findings, the Japanese medical fee system has incorporated the requirements for multidisciplinary conferences into various reimbursement categories, such as the Additions for Discharge Support, Early Nutritional Intervention Management, and Home-visit Pressure Ulcer Management [7, 8]. Furthermore, the Guidelines for Ward Services by Pharmacists Ver. 1.2 [9] and Guidelines for Drug Information Services 2018 [10] published by the Japan Society of Hospital Pharmacists emphasize the importance of conferences, both among multidisciplinary teams and within the pharmacy department itself.

However, such conferences are not fully established in Japan. A nationwide survey by Yano et al [11]. for the Ministry of Health, Labor, and Welfare research group reported that only 0.4% of facilities conduct daily conferences, and no reports validate their utility. Although the benefits of multidisciplinary conferences are becoming increasingly evident globally, no studies have explored the specific outcomes of inter-pharmacist conferences, where cases are routinely shared and discussed with a focus on pharmacotherapy.

In practice, maintaining inter-pharmacist conferences within daily clinical routines is challenging. Several barriers have been reported, including heavy workloads, difficulty in securing participants, and lack of clarity regarding objectives and procedures [12]. Therefore, identifying the factors that enabled the continuation of inter-pharmacist conferences for over a year and the outcomes achieved during that process could provide valuable insights for further development of pharmacy practices.

Following the 2022 revision of the Model Core Curriculum for Pharmaceutical Education in Japan, Kameda Medical Center (Kamogawa, Japan) implemented daily inter-pharmacist conferences since November 2023 to support postgraduate pharmacist education, in addition to multidisciplinary conferences. This initiative aims to not only solve patient-specific clinical problems and optimize drug therapy but also to standardize perspectives on pharmaceutical evaluation and intervention. This is achieved through activities such as knowledge sharing regarding drug selection and reviewing and refining inquiries and prescription proposals. These activities are expected to improve the clinical judgement of pharmacists, activate information sharing, and expand their contributions to pharmacotherapy within the medical team.

Accordingly, this study aimed to evaluate the perceived utility of daily inter-pharmacist conferences conducted continuously for one year at Kameda Medical Center in relation to pharmacists’ individual growth and clinical practice. The study also aimed to explore factors associated with their continuity. Through this, the study ultimately aims to provide foundational knowledge for the dissemination and establishment of inter-pharmacist conferences and contribute to the qualitative improvement of ward pharmacy services.

## Methods

### Study design

This was a cross-sectional study using a questionnaire-based survey.

### Implementation of inter-pharmacist case conferences

**Fig. 1:**
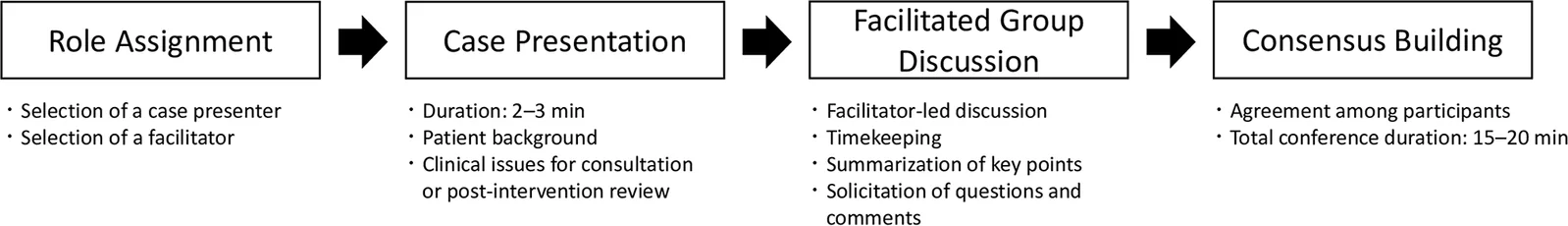
Workflow of the pharmacist case conference. Each conference group comprised 5–10 pharmacists. Among the participants, one pharmacist was assigned as the case presenter and another as the facilitator. The case presenter delivered a brief presentation (2–3 min) describing the patient characteristics and the clinical issues to be discussed, including cases requiring consultation during routine practice or a retrospective review of prior interventions. The facilitator guided the discussion by managing the time, summarizing key points, and encouraging questions and comments from all participants. The conference proceeded until a consensus was reached among the participants, with a total session duration of approximately 15–20 min

In November 2023, the Kameda Medical Center (Kamogawa, Japan) launched daily inter-pharmacist case conferences to discuss pharmacological issues related to the patients under their care. Participants were divided into three groups based on their clinical specialty area, with approximately 5–10 pharmacists participating per group. The implementation of the case conferences is shown in Fig. 1. At each conference, a presenter and facilitator were selected from among the participating pharmacists to discuss cases requiring consultation during the duties of that day or to reflect on cases where interventions had already been completed.

Prior to participation, all pharmacists attended lectures covering the objectives of the conference, the operational flow, case presentation methods, and the One-minute Preceptor model as a mindset for participation. Educational materials for these sessions were developed by board-certified senior pharmacotherapy specialists and preceptors for pharmacy practice experience.

During the conferences, case presentations covering patient backgrounds and points for discussion were limited to 2–3 min. The facilitator was responsible for time management, summarizing the presentation, and soliciting questions and comments from other participants. Each session lasted 15–20 min, including the time taken to reach a consensus among the participants.

### Questionnaire development

As no standardized tool exists to evaluate the utility of inter-pharmacist case conferences, a questionnaire based on the Attention, Relevance, Confidence, Satisfaction model was developed, which is a motivational model designed to enhance the desire to learn [13].

The questionnaire comprised 16 items. Items No. 1 and 2 were binary (Yes/No) questions regarding experience, whereas items No. 3–16 were evaluated using a 7-point Likert scale that ranged from 1 (strongly disagree/lowest) to 7 (strongly agree/highest). In addition to these items, three free-text questions were included to collect participants’ views on factors supporting continuation, factors that could reduce willingness to participate, and requests for improvement. In education, learning through practical experience is more impactful than merely acquiring knowledge [14]. Therefore, this study focused on the potential contribution of practical roles to the educational effect and included items to identify whether the participants had experience as facilitators or case presenters. Furthermore, considering that environments with psychological safety encourage active participation in group discussions [15, 16], items to assess psychological safety were also incorporated. Additionally, an item regarding the intention to continue was included to investigate the association between these factors and the sustainability of the conferences.

The content validity of the questionnaire was ensured through a consensus-building process among four pharmacists, including a board-certified senior oncology pharmacist and a board-certified senior pharmacotherapy specialist, each with over five years of clinical experience. The survey was administered using Microsoft Forms (Table 1). The free-text responses were reviewed descriptively to contextualize the quantitative findings and identify recurring implementation-related themes.

**Table 1:** Questionnaire items to determine the utility of inter-pharmacist case conferences

Item No.	Question (Abbreviated label)	Response Scale
Q1	Have you ever served as a facilitator in an inter-pharmacist case conference? (Facilitator experience)	Yes/No
Q2	Have you ever presented a case in an inter-pharmacist case conference? (Case presenter experience)	Yes/No
Q3	Were you satisfied with your case presentation in the inter-pharmacist case conference? (Satisfaction with presentation)	7-point Likert
Q4	Did presenting a case in the conference increase your confidence in your pharmaceutical judgement? (Confidence in judgement)	7-point Likert
Q5	Do you feel that you have grown as a pharmacist by presenting a case, compared to before the presentation? (Sense of professional growth)	7-point Likert
Q6	Do you feel that the discussions in the conference can be applied to your own clinical judgement and differentiation? (Utility in clinical judgement)	7-point Likert
Q7	Do you feel that the knowledge and experience gained from the conference can be applied to other cases? (Generalizability to other cases)	7-point Likert
Q8	Do you think that discussing cases in the conference improves the quality of drug therapy compared to not holding such conferences? (Improvement in therapy quality)	7-point Likert
Q9	Did participating in the conference increase your interest in deeply reflecting on cases from medical and pharmaceutical perspectives? (Interest in case reflection)	7-point Likert
Q10	Have you started utilizing the case conferences when you face difficulties in clinical judgement or differentiation? (Conference utilization for judgement)	7-point Likert
Q11	Has the frequency of opportunities to discuss or consult about cases with colleagues increased compared to before participating in the conferences? (Increased consultation frequency)	7-point Likert
Q12	Have you utilized the knowledge and skills learned in the conferences for instructing colleagues, juniors, or students? (Knowledge transfer to others)	7-point Likert
Q13	Do you feel that you can freely express your opinions during the inter-pharmacist case conferences? (Freedom to express opinions)	7-point Likert
Q14	Do you feel that the case conferences provide opportunities to utilize your specialty or areas of expertise? (Opportunity for expertise)	7-point Likert
Q15	Are you satisfied with the facilitation and the way discussions are conducted in the conferences? (Satisfaction with facilitation)	7-point Likert
Q16	Would you like to continue participating in the inter-pharmacist case conferences in the future? (Motivation for continuity)	7-point Likert

### Study participants

Out of the 92 pharmacists employed at Kameda Medical Center, the survey was distributed to 54 pharmacists who provided consent. Of these, 40 pharmacists who had participated in inter-pharmacist case conferences at least once were included in the final analyses.

### Study period

The survey was conducted in March 2025, 16 months (one year and four months) after the initiation of the inter-pharmacist case conferences.

### Outcome measures

The primary endpoint of this study was to identify and clarify the factors constituting the utility of inter-pharmacist case conferences by performing an exploratory factor analysis (EFA) on questionnaire response data.

The secondary endpoints were as follows:

To identify the factors contributing to long-term sustainability through multiple regression analysis using the mean scores of the factors extracted from the EFA as explanatory variables and the motivation for conference continuity as the objective variable.

To evaluate the impact of experience as a facilitator or case presenter on the factors identified through factor analysis.

### Statistical analysis

Descriptive statistics were obtained by calculating the medians and interquartile ranges for continuous variables and frequency distributions for categorical variables. Means and standard deviations were additionally calculated for each questionnaire item and are presented in the supplementary material. EFA was conducted to explore the latent structure of the utility of inter-pharmacist case conferences. The principal factor method was used for extraction, with squared multiple correlations as the initial communality estimates. The number of factors was determined based on the scree plot shape and Kaiser’s criterion (eigenvalues > 1.0). The number of factors was also evaluated comprehensively based on cumulative explained variance and interpretability of the factor structure. Varimax orthogonal rotation was applied. Factor loadings ≥ 0.40 were considered significant for interpretation, and items loading ≥ 0.40 on multiple factors were assigned to the factor with the highest loading. The extracted factors were named according to their constituent contents. Cronbach’s α and corrected item-total correlations were calculated for each extracted factor to assess internal consistency. Corrected item-total correlations were calculated as Pearson’s correlation coefficients between each item and the mean score of the remaining items within the same factor.

For the secondary analysis, a multiple regression analysis was performed to examine the association between factor scores and motivation for conference continuity (item 16). The motivation for continuity was set as the dependent variable, with the extracted factor scores as independent variables. The explanatory variables were limited a priori to the three factor scores extracted by EFA. No stepwise or other data-driven variable selection procedures were used. To reduce the risk of overfitting given the limited sample size, the number of explanatory variables was restricted to these three theoretically and empirically derived factors. Residual distributions were inspected using predicted-versus-residual and Q–Q plots to evaluate assumptions regarding linearity, homoscedasticity, and normality. The normality of residuals was assessed using the Shapiro-Wilk test. Multicollinearity was assessed using the variance inflation factor (VIF), with a VIF < 3 considered acceptable.

Furthermore, a two-way analysis of variance (ANOVA) was conducted to evaluate the effects of facilitator and case presenter experiences on the factor scores. Although the initial plan included examining interactions, the analysis focused on the main effects of each experience owing to the limited sample size. The homogeneity of variance was assessed using Levene’s test, and all statistical analyses were performed using JMP Pro version 17 (SAS Institute Inc., Cary, NC, USA). The significance level was set at two-tailed *p* < 0.05. When performing ANOVA on multiple factor scores, the significance level was adjusted using the Bonferroni correction to control for Type I error inflation.

### Ethical considerations

This study was approved by the Institutional Review Board of the Kameda Medical Center (Approval No. 25–030). The purpose of the study was explained to all respondents, and informed consent was obtained prior to their participation.

## Results

Between November 1, 2023 and October 31, 2024, 348 inter-pharmacist case conferences were conducted. The recovery rate of the questionnaires was 58.7% (54/92), with a 100% response rate from those who engaged. The final analysis included data from 40 pharmacists. In terms of their years of experience as pharmacists, 7 (17.5%) had 1–2 years, 9 (22.5%) had 3–5 years, 12 (30%) had 6–10 years, and 12 (30%) had ≥ 11 years.

### Questionnaire on inter-pharmacist conferences

**Fig. 2:**
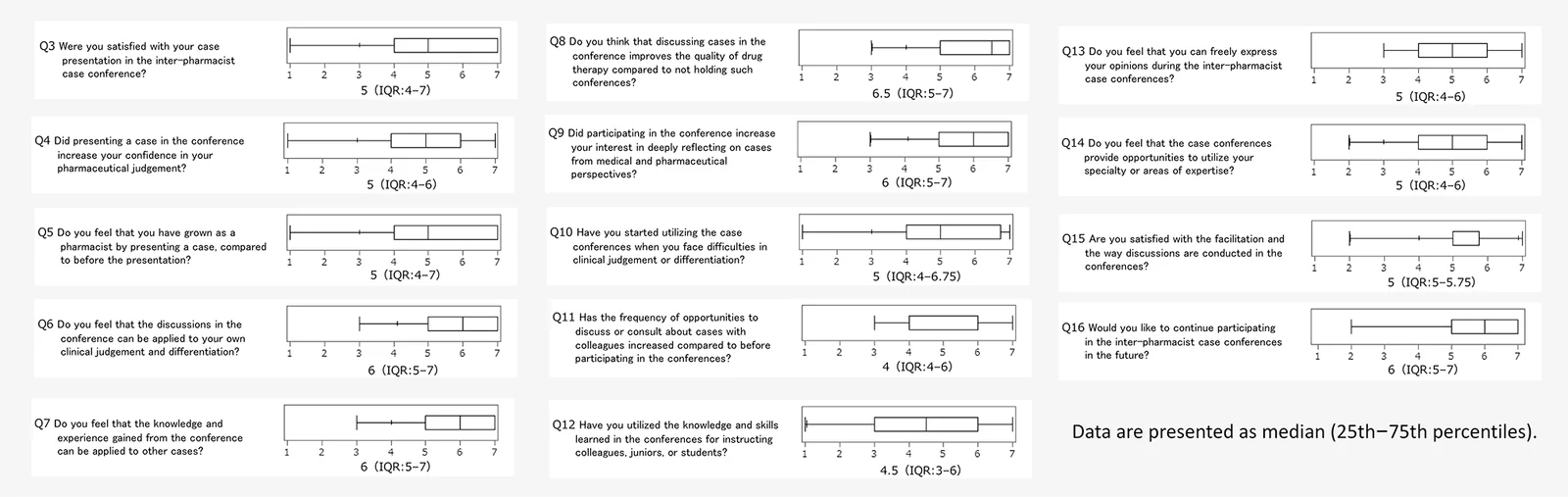
Results of the 16-item questionnaire regarding the utility of inter-pharmacist conferences. Data are presented as box-and-whisker plots representing a 7-point Likert scale (1 = strongly disagree/lowest, 7 = strongly agree/highest). Values below each plot indicate the median and interquartile range (25th–75th percentiles). Some items showed mild skewness or ceiling tendencies; therefore, means and standard deviations are shown in Supplementary table S1

Box-and-whisker plots for 14 items (Q3–Q16) are shown in Fig. 2. The median scores for all items were ≥ 5 except for Q11 (increased consultation frequency; median: 4) and Q12 (knowledge transfer to others; median: 4.5). Notably, high median scores were observed for Q8 (improvement in therapy quality; median = 6.5) and Q9 (interest in case reflection; median = 6). By contrast, Q11 and Q12 tended to have lower scores than those of the other items, with medians of ~4. The means and standard deviations for each questionnaire item are shown in Supplementary Table S1.

In terms of Q16 (motivation for continuity), the median score was 6, and 82.5% (33/40) of participants expressed a desire to continue the conferences in the future. Free-text responses provided additional descriptive information regarding the sustainability of the case conferences. Frequently mentioned factors supporting continuation included integration into routine working hours, a brief and low-burden format, psychological safety, and opportunities to receive advice on difficult cases. In contrast, factors that could reduce willingness to participate included forced participation or case presentation, prolonged sessions, insufficient staffing coverage, repeated case presentation by the same individuals, and difficulty participating from non-ward departments or online settings.

### Primary outcome: factor structure of the utility of inter-pharmacist conferences

The exploratory factor analysis identified three factors with a cumulative contribution rate of 75.5% (Table 2). The eigenvalues, scree plot, and final communality estimates are shown in Supplementary Tables S2 and S3 and Supplementary Figure S1.

**Table 2:** Exploratory factor analysis results (factor loadings after rotation)

Item	Factor 1	Factor 2	Factor 3
Q5 Sense of professional growth	**0.86**	0.29	0.19
Q4 Confidence in judgement	**0.85**	0.25	0.32
Q3 Satisfaction with presentation	**0.85**	0.21	0.21
Q10 Conference utilization for judgement	**0.69**	0.15	0.42
Q6 Utility in clinical judgement	0.28	**0.87**	0.18
Q7 Generalizability to other cases	0.23	**0.87**	0.37
Q8 Improvement in therapy quality	0.27	**0.81**	0.29
Q9 Interest in case reflection	0.16	**0.69**	0.42
Q13 Freedom to express opinions	0.19	0.25	**0.78**
Q15 Satisfaction with facilitation	0.35	0.28	**0.69**
Q12 Knowledge transfer to others	0.30	0.31	**0.65**
Q14 Opportunity for expertise	0.33	0.43	**0.62**
Q11 Increased consultation frequency	0.48	0.36	**0.49**

Note: Factor extraction was performed using the principal factor method followed by varimax rotation with Kaiser normalization. The values are rounded to two decimal places. For each item, the highest factor loading greater than 0.40 is indicated in bold

Factor 1 comprised Q5 (sense of professional growth), Q4 (confidence in judgement), Q3 (satisfaction with presentation), and Q10 (conference utilization for judgement). These items reflected the aspects of self-efficacy and professional development experienced by pharmacists at conferences. Thus, Factor 1 was interpreted as confidence and professional growth through inter-pharmacist conferences.

Factor 2 consisted of Q6 (utility in clinical judgement), Q7 (generalizability to other cases), Q8 (improvement in therapy quality), and Q9 (interest in case reflection). As these items relate to the enhancement of clinical judgement and quality of drug therapy, Factor 2 was designated as improvement in clinical judgement and quality of drug therapy.

Factor 3 included Q13 (freedom to express opinions), Q15 (satisfaction with facilitation), Q12 (knowledge transfer to others), Q14 (opportunity for expertise), and Q11 (increased consultation frequency). These items reflect the promotion of communication among pharmacists, fulfillment of educational roles, and improvement of the environment for expressing opinions. Consequently, Factor 3 was interpreted as promotion of inter-pharmacist communication and improvement of the environment for expressing opinions (Table 3).

**Table 3:** Names, interpretations, and mean scores of study factors

Factor	Name (Abbreviation)	Interpretation	Mean Score
Factor 1	Confidence and professional growth through inter-pharmacist conferences (Professional growth)	Perceived gains in confidence in pharmaceutical judgement and personal growth through case presentations and discussions.	5.09
Factor 2	Improvement in clinical judgement and quality of drug therapy (Clinical judgement)	Perceived usefulness for supporting clinical decision-making skills and the qualitative improvement of drug therapy.	5.90
Factor 3	Promotion of inter-pharmacist communication and improvement of the environment for expressing opinions (Workplace environment)	Aspects related to intra-departmental communication, educational roles, and the environment for expressing opinions.	4.87

Note: Mean scores are calculated based on a 7-point Likert scale (1: lowest, 7: highest)

In terms of the mean scores for each factor, Factor 2 (improvement in clinical judgement and quality of drug therapy) showed the highest score (mean: 5.90), followed by Factor 1 (confidence and professional growth through inter-pharmacist conferences; mean: 5.09) and Factor 3 (promotion of inter-pharmacist communication and improvement of the environment for expressing opinions; mean: 4.87).

The internal consistency of each factor was high, with Cronbach’s α values of 0.94 for Factor 1, 0.95 for Factor 2, and 0.89 for Factor 3. Corrected item-total correlations within each factor ranged from 0.70 to 0.92, indicating acceptable item consistency. These descriptive statistics and internal consistency indices are shown in Supplementary Table S1.

### Secondary outcome 1: Association between factors and the motivation for conference continuity

A multiple regression analysis was performed with the motivation for conference continuity as the dependent variable and the three factors (professional growth, Factor 1; clinical judgement, Factor 2; and workplace environment, Factor 3) as independent variables (Table 4). The coefficient of determination (R^2^) was 0.561 (*p* < 0.001), indicating that the model was statistically significant. Individually, professional growth (β = 0.357, *p* = 0.016) and clinical judgement (β = 0.646, *p* = 0.003) were significantly associated with the motivation for continuity. In contrast, workplace environment showed no significant association (*p* = 0.821). The VIF for each variable ranged from 1.95 to 2.67, thereby indicating no concerns regarding multicollinearity. Residual diagnostics were performed for the multiple regression model. The residual-versus-predicted plot did not show marked heteroscedasticity or a clear systematic pattern. However, the Q–Q plot suggested mild deviations from normality at the tails, and the Shapiro-Wilk test indicated a statistically significant deviation from normality (W = 0.93, *p* = 0.013). These diagnostic results are shown in Supplementary Figure S2 and Supplementary Table S4.

**Table 4:** Association between factors and the motivation for conference continuity (multiple regression analysis)

Independent Variable	Estimate	Standard Error	t-ratio	*p*-value	VIF
Factor 1: Professional Growth	0.36	0.14	2.5	0.02^*^	1.95
Factor 2: Clinical Judgement	0.65	0.20	3.2	<0.01^*^	1.98
Factor 3: Workplace Environment	−0.05	0.22	−0.2	0.82	2.67

Note. Dependent variable: motivation for conference continuity (item 16). R^2^ = 0.561, *p* < 0.001. VIF: variance inflation factor. Asterisks (*) indicate statistical significance at *p* < 0.05

### Secondary outcome 2: Impact of facilitator and case presenter experience on factor scores

The results of the two-way ANOVA are presented in Table 5. For Factor 1 (professional growth), a significant main effect was observed for case presenter experience (*p* < 0.01), with those with experience as presenters showing significantly higher factor scores. To account for the use of three separate factor scores in the analysis, the significance level was adjusted using the Bonferroni correction to *p* < 0.017. The association between Factor 1 and case presenter experience remained significant after this correction. In terms of Factor 2 (clinical judgement) and Factor 3 (workplace environment), no pronounced main effects were found for either facilitator experience or case presenter experience. The interaction effects were not analyzed owing to constraints related to the degrees of freedom.

**Table 5:** Impact of facilitator and case presenter experience on each factor score (two-way ANOVA)

Factor	Main Effect (Facilitator Experience) *p*-value	Main Effect (Case Presenter Experience) *p*-value
Factor 1: Professional Growth	0.59	<0.01*
Factor 2: Clinical Judgement	0.67	0.68
Factor 3: Workplace Environment	0.77	0.14

Note: The significance level was adjusted to *p* < 0.017 using Bonferroni correction to account for the analysis of the three factors. The asterisk (*) indicates statistical significance after correction. Interaction effects were excluded from the analysis owing to the limited degrees of freedom. ANOVA, analysis of variance

## Discussion

This study investigated the perceived utility of inter-pharmacist case conferences. Based on the questionnaire results, the utility of these conferences was categorized into the following three factors: professional growth, clinical judgement, and workplace environment. Notably, the factor score for clinical judgement was the highest. This suggests that inter-pharmacist case conferences may be perceived as particularly useful for supporting clinical judgement in daily practice. Furthermore, high scores in individual items, such as improvement in therapy quality and interest in case reflection, indicate that these conferences may function as a practice-based learning opportunity directly linked to daily clinical practice.

In contrast, the median scores for increased consultation frequency and knowledge transfer to others were relatively low. This suggests room for improvement in environmental aspects, such as knowledge sharing and consultation frameworks. These findings are consistent with the lower score for Factor 3 (workplace environment) than for the other factors.

Multiple regression analysis showed that professional growth and clinical judgement were significantly associated with motivation for conference continuity. These findings suggest that pharmacists who perceived greater professional growth and practical usefulness in clinical judgement tended to report stronger motivation to continue participating in the conferences. Therefore, perceived growth and applicability to clinical judgement may be important elements related to the sustainability of inter-pharmacist case conferences. Furthermore, the secondary analysis showed that participants with experience as case presenters had higher scores for professional growth. As case presentation may encourage self-regulated and active learning, intentionally providing such opportunities may be a useful strategy for supporting pharmacist education.

Based on these findings, several practical strategies may support the sustainability of inter-pharmacist case conferences. First, providing structured and rotating opportunities for case presentation may help participants experience professional growth more consistently. Second, maintaining a brief and standardized format, such as short case presentations followed by facilitated discussion, may reduce the operational burden and facilitate integration into routine work. Third, training facilitators to summarize key discussion points and encourage participation may help maintain psychological safety and discussion quality. Finally, because the scores for increased consultation frequency and knowledge transfer to others were relatively low, post-conference sharing tools, such as brief summaries of learning points or examples of prescription proposals, may be useful for promoting knowledge transfer beyond the conference itself.

These free-text responses may help explain the relatively lower scores for increased consultation frequency and knowledge transfer to others. They also suggest that sustainable implementation requires not only educational value but also operational support, equitable participation opportunities, and mechanisms for sharing learning points after the case conference.

Despite these insights, this study has inherent limitations related to its single-center design, sample size, and reliance on subjective assessments. First, this was a single-center study conducted at Kameda Medical Center, a large-scale acute care hospital. Consequently, the findings may have been influenced by institution-specific factors, including the unique conference format, staffing structure, educational culture, and management support. Furthermore, the analysis was limited to 40 participating pharmacists who provided informed consent and had experience attending the case conferences. This small sample size may have introduced selection bias, as pharmacists who were more interested in clinical learning, case discussion, or professional development may have been more likely to participate and respond. Hence, caution is required when generalizing these results to pharmacists who did not participate in the conferences or to facilities with different sizes, staffing models, or organizational cultures.

Second, the psychometric properties of the original questionnaire, such as construct validity and test-retest reliability, have not been sufficiently validated. Although Cronbach’s α and corrected item-total correlations were additionally calculated in this study, further validation is needed. In addition, the high median scores suggest a potential ceiling effect, which may have limited the sensitivity of the statistical analysis. Because of the limited sample size, cross-validation and bootstrap analyses to assess the stability of the factor structure were not performed. Similarly, stratified sensitivity analyses and structural equation modeling were not performed, as these analyses would require larger samples to obtain stable estimates. Therefore, further multicenter studies are necessary to confirm the reproducibility of the identified factor structure.

Third, operational factors that may influence the sustainability of the case conferences were not fully evaluated. These factors include workload, staffing levels, ward assignment, timing of the conferences, facilitator skills, accessibility from non-ward departments or online settings, and the degree of managerial support. Because such factors may affect both participation and perceived usefulness, future studies should incorporate these operational variables to clarify the conditions under which inter-pharmacist case conferences can be sustainably implemented.

Fourth, the outcomes were restricted to subjective self-assessment. Objective indicators, such as prescription proposal rates or clinical patient outcomes, were not collected; therefore, the extent of actual behavioral changes among pharmacists remains unclear. To address these limitations, future prospective multicenter studies utilizing objective endpoints and patient outcomes are required to verify the actual clinical impact of inter-pharmacist case conferences.

Finally, free-text responses were reviewed descriptively to contextualize the quantitative findings; however, a formal qualitative analysis, such as independent coding by multiple researchers or interview-based thematic analysis, was not conducted. Future mixed-methods studies may help clarify the contextual factors underlying the lower-scoring items and the implementation barriers identified in this study.

## Conclusions

This study identified factors associated with the perceived utility of inter-pharmacist case conferences and examined factors related to their sustainability. These findings suggest that focusing on clinical judgement and providing opportunities for case presentations may be important for the ongoing professional development of pharmacists. Accordingly, future research should involve multicenter collaborative studies to collect robust data and conduct further investigations using objective patient outcomes and implementation-related indicators as endpoints to verify the clinical impact and sustainability of these conferences.

## Electronic supplementary material

Below is the link to the electronic supplementary material.

Supplementary material 1

Supplementary material 2

Supplementary material 3

Supplementary material 4

Supplementary material 5

Supplementary material 6

## Data Availability

The datasets used and/or analysed during the current study are available from the corresponding author on reasonable request.

## Abbreviations

EFA: Exploratory factor analysis
VIF: Variance inflation factor
ANOVA: Analysis of variance.
