# Supplementary material for: An evaluation of factors affecting the usefulness and sustainability of inter-pharmacist case conferences
Source: J Pharm Health Care Sci. 2026 Jun 15;12:77. doi: 10.1186/s40780-026-00599-7 (PMC13495269; doi:10.1186/s40780-026-00599-7)
Supplement: Supplementary file 1 [file 40780_2026_599_MOESM1_ESM.docx]

**Supplementary Figure S1. Scree plot of the exploratory factor analysis.**

The scree plot supported a three-factor solution, consistent with Kaiser’s criterion eigenvalues >1.0 and the interpretability of the extracted factors.
