# Supplementary material for: An evaluation of factors affecting the usefulness and sustainability of inter-pharmacist case conferences
Source: J Pharm Health Care Sci. 2026 Jun 15;12:77. doi: 10.1186/s40780-026-00599-7 (PMC13495269; doi:10.1186/s40780-026-00599-7)
Supplement: Supplementary file 2 [file 40780_2026_599_MOESM2_ESM.docx]

**Supplementary Figure S2A. Residuals versus predicted values plot for the multiple regression model.**

Residuals were plotted against predicted values to visually assess linearity and homoscedasticity.

**Supplementary Figure S2B. Normal Q–Q plot of residuals for the multiple regression model.**

The normal Q–Q plot was used to visually assess the normality of residuals.
