# Supplementary material for: An evaluation of factors affecting the usefulness and sustainability of inter-pharmacist case conferences
Source: J Pharm Health Care Sci. 2026 Jun 15;12:77. doi: 10.1186/s40780-026-00599-7 (PMC13495269; doi:10.1186/s40780-026-00599-7)
Supplement: Supplementary file 3 [file 40780_2026_599_MOESM3_ESM.docx]

**Supplementary Table S1. Descriptive statistics and internal consistency of questionnaire items**

| Factor | Item No. | Abbreviated label | Mean ± SD | Cronbach’s α for factor | Corrected item-total correlation |
| --- | --- | --- | --- | --- | --- |
| Factor 1: Confidence and professional growth through inter-pharmacist conferences | Q3 | Satisfaction with presentation | 5.13 ± 1.67 | 0.94 | 0.85 |
|  | Q4 | Confidence in judgement | 5.03 ± 1.56 | 0.94 | 0.90 |
|  | Q5 | Sense of professional growth | 5.20 ± 1.68 | 0.94 | 0.88 |
|  | Q10 | Conference utilization for judgement | 5.00 ± 1.68 | 0.94 | 0.76 |
| Factor 2: Improvement in clinical judgement and quality of drug therapy | Q6 | Utility in clinical judgement | 5.78 ± 1.12 | 0.95 | 0.87 |
|  | Q7 | Generalizability to other cases | 5.83 ± 1.24 | 0.95 | 0.92 |
|  | Q8 | Improvement in therapy quality | 6.05 ± 1.15 | 0.95 | 0.89 |
|  | Q9 | Interest in case reflection | 5.93 ± 1.07 | 0.95 | 0.80 |
| Factor 3: Promotion of inter-pharmacist communication and improvement of the environment for expressing opinions | Q11 | Increased consultation frequency | 4.80 ± 1.42 | 0.89 | 0.70 |
|  | Q12 | Knowledge transfer to others | 4.55 ± 1.50 | 0.89 | 0.72 |
|  | Q13 | Freedom to express opinions | 5.13 ± 1.36 | 0.89 | 0.74 |
|  | Q14 | Opportunity for expertise | 4.85 ± 1.33 | 0.89 | 0.75 |
|  | Q15 | Satisfaction with facilitation | 5.03 ± 1.07 | 0.89 | 0.78 |
| Outcome item | Q16 | Motivation for continuity | 5.60 ± 1.36 | N/A | N/A |

Note: Items were rated on a 7-point Likert scale. Cronbach’s α was calculated for each extracted factor. Corrected item-total correlations were calculated as Pearson’s correlation coefficients between each item and the mean score of the remaining items within the same factor. Q16 was used as the outcome variable in the multiple regression analysis and was not included in the exploratory factor analysis or internal consistency analysis.
