# Supplementary material for: An evaluation of factors affecting the usefulness and sustainability of inter-pharmacist case conferences
Source: J Pharm Health Care Sci. 2026 Jun 15;12:77. doi: 10.1186/s40780-026-00599-7 (PMC13495269; doi:10.1186/s40780-026-00599-7)
Supplement: Supplementary file 4 [file 40780_2026_599_MOESM4_ESM.docx]

**Supplementary Table S2. Eigenvalues and explained variance used to determine the number of factors**

| Factor number | Eigenvalue | Proportion of variance (%) | Cumulative variance (%) |
| --- | --- | --- | --- |
| 1 | 7.89 | 60.71 | 60.71 |
| 2 | 1.58 | 12.17 | 72.88 |
| 3 | 1.04 | 8.03 | 80.90 |
| 4 | 0.62 | 4.78 | 85.68 |
| 5 | 0.46 | 3.53 | 89.21 |
| 6 | 0.32 | 2.43 | 91.64 |
| 7 | 0.29 | 2.22 | 93.86 |
| 8 | 0.27 | 2.04 | 95.90 |
| 9 | 0.16 | 1.26 | 97.16 |
| 10 | 0.13 | 1.01 | 98.17 |
| 11 | 0.10 | 0.79 | 98.96 |
| 12 | 0.08 | 0.62 | 99.58 |
| 13 | 0.05 | 0.42 | 100.00 |

Note: The number of factors was determined based on Kaiser’s criterion, the scree plot, cumulative explained variance, and interpretability of the factor structure.
