# Supplementary material for: An evaluation of factors affecting the usefulness and sustainability of inter-pharmacist case conferences
Source: J Pharm Health Care Sci. 2026 Jun 15;12:77. doi: 10.1186/s40780-026-00599-7 (PMC13495269; doi:10.1186/s40780-026-00599-7)
Supplement: Supplementary file 5 [file 40780_2026_599_MOESM5_ESM.docx]

**Supplementary Table S3. Final communalities**

| Item No. | Abbreviated label | Final communality |
| --- | --- | --- |
| Q3 | Satisfaction with presentation | 0.80 |
| Q4 | Confidence in judgement | 0.89 |
| Q5 | Sense of professional growth | 0.86 |
| Q6 | Utility in clinical judgement | 0.87 |
| Q7 | Generalizability to other cases | 0.94 |
| Q8 | Improvement in therapy quality | 0.81 |
| Q9 | Interest in case reflection | 0.68 |
| Q10 | Conference utilization for judgement | 0.68 |
| Q11 | Increased consultation frequency | 0.59 |
| Q12 | Knowledge transfer to others | 0.61 |
| Q13 | Freedom to express opinions | 0.71 |
| Q14 | Opportunity for expertise | 0.68 |
| Q15 | Satisfaction with facilitation | 0.68 |

Note: Communalities indicate the proportion of variance in each item explained by the extracted factors.
