# Supplementary material for: An evaluation of factors affecting the usefulness and sustainability of inter-pharmacist case conferences
Source: J Pharm Health Care Sci. 2026 Jun 15;12:77. doi: 10.1186/s40780-026-00599-7 (PMC13495269; doi:10.1186/s40780-026-00599-7)
Supplement: Supplementary file 6 [file 40780_2026_599_MOESM6_ESM.docx]

**Supplementary Table S4. Normality test of residuals from the multiple regression model**

| Model | Test | Statistic | p-value |
| --- | --- | --- | --- |
| Multiple regression model for motivation for continuity | Shapiro-Wilk test | W = 0.93 | 0.013 |

Note: The normality of residuals from the multiple regression model was assessed using the Shapiro-Wilk test.
